# Arrhythmic Mitral Valve Prolapse and Mitral Annular Disjunction: Clinical Features, Pathophysiology, Risk Stratification, and Management

**DOI:** 10.3390/jcdd9020061

**Published:** 2022-02-16

**Authors:** Apurba K. Chakrabarti, Frank Bogun, Jackson J. Liang

**Affiliations:** Section of Electrophysiology, Division of Cardiology, Department of Internal Medicine, University of Michigan, Ann Arbor, MI 48109, USA; akchakra@med.umich.edu (A.K.C.); fbogun@med.umich.edu (F.B.)

**Keywords:** mitral valve prolapse, mitral annular disjunction, sudden cardiac death, catheter ablation, implantable cardiac defibrillator, multi-modality imaging

## Abstract

Mitral valve prolapse (MVP) is a common cause of valvular heart disease. Although many patients with MVP have a benign course, there is increasing recognition of an arrhythmic phenotype associated with ventricular arrhythmias and sudden cardiac death (SCD). Pathophysiologic mechanisms associated with arrhythmias include cardiac fibrosis, mechanical stress induced changes in ventricular refractory periods, as well as electrophysiologic changes in Purkinje fibers. Clinically, a variety of risk factors including demographic, electrocardiographic, and imaging characteristics help to identify patients with MVP at the highest at risk of SCD and arrhythmias. Once identified, recent advances in treatment including device therapy, catheter ablation, and surgical interventions show promising outcomes. In this review, we will summarize the incidence of ventricular arrhythmias and SCD in patients with MVP, the association with mitral annular disjunction, mechanisms of arrhythmogenesis, methods for arrhythmic and SCD risk stratification including findings with multimodality imaging, and treatments for the primary and secondary prevention of SCD.

## 1. Introduction

Mitral valve prolapse (MVP) is the abnormal systolic excursion of mitral valve leaflets into the left atrium. First described clinically in the 1960s with cine-angiocardiography [1], it is now defined as at least 2 mm of superior displacement of mitral valve leaflets above the mitral annulus, measured echocardiographically in the parasternal or apical long axis views. Using modern definitions, MVP has a prevalence between 1 and 3% [2,3,4]. MVP is among the most common causes of chronic primary mitral regurgitation, although only a minority of patients will progress to severe mitral regurgitation [5].

Pathologically, MVP is a spectrum of anatomical abnormalities ranging from the classic form (Barlow’s Disease) with thickened redundant leaflets to a non-classic form (fibroelastic deficiency) with thinner leaflets [6,7]. In classic MVP, myxoid infiltration results in leaflet thickening ≥ 5 mm, bileaflet prolapse with redundant and billowing leaflets, mitral annular dilation, and chordal changes. In contrast, non-classic MVP is associated with leaflet thinning due to reduced connective tissue production and more often has segmental prolapse [7]. MVP can be a primary abnormality or secondary to another syndrome. Primary MVP is typically sporadic, but there are familial/genetic patterns. Several genes (FLNA, DCHS1, DZIP1, and PLD1) with a wide variety of inheritance patterns have been associated with familial MVP and techniques such as exome sequencing and genome-wide association studies are identifying novel genes as well [7,8,9]. FLNC mutations are associated with an arrhythmogenic form of MVP [10]. Alternatively, MVP can be secondary to connective tissue syndromes (e.g., Marfan, Ehlers–Danlos, Loeys–Dietz) or a variety of other cardiac disorders. 

MVP is related to but distinct from mitral annular disjunction (MAD). The mitral annulus is an anatomically “D”-shaped structure, into which the anterior and posterior mitral valve leaflets insert [11,12]. MAD is defined as mitral annular detachment from the basal left ventricular (LV) myocardium with an abnormal systolic excursion of the leaflet hinge point into the left atrium. Pathologically, MAD was first described in the context of MVP in the 1980s, but MAD has been observed in the absence of MVP [13,14,15,16]. Abnormal systolic curling of the posterior mitral annulus, which was first described in MVP patients in the 1970s, has recently been shown to strongly correlate with MAD [17,18]. The displacement distance can be measured non-invasively by echocardiography, cardiac computed tomography (CT), and cardiac magnetic resonance (CMR). In subsequent studies of various cohorts of MVP, MAD has been identified in approximately 15–55% of patients with significant variation based on the different cohorts studied and imaging modalities utilized [19,20,21,22,23,24,25,26,27]. A systematic review found MAD in about 33% of MVP patients [19].

The long-term outcome of patients with MVP is heterogenous [2,5,28]. While many patients do well, there is increasing recognition of a subgroup of patients with an arrhythmic course involving ventricular arrhythmias (VA) and sudden cardiac death (SCD) [4,29,30,31]. Given the relatively high prevalence of MVP, the identification and management of MVP patients at risk of SCD is critical. 

In this review, we will summarize the mechanisms of arrhythmogenesis, incidence of SCD in patients with MVP, methods for SCD risk stratification including novel findings with multimodality imaging, and treatments for the primary and secondary prevention of SCD.

## 2. Arrhythmogenesis

**Figure 1:**
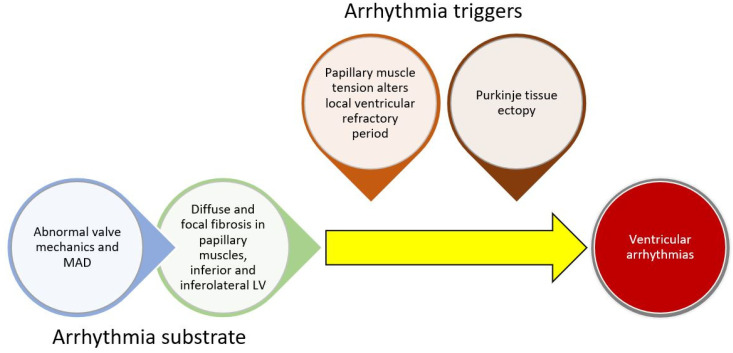
Conceptual framework for ventricular arrhythmogenesis involving the development of cardiac substrate and triggers for arrhythmias.

While poorly understood, the current understanding of arrhythmogenesis in MVP involves the development of a substrate for arrhythmias (cardiac fibrosis) combined with a trigger for arrhythmias (Figure 1). The anatomic defect of MVP, leaflet prolapse, causes abnormal tension on the papillary muscles and abnormal wall stress on adjacent LV basal and inferolateral myocardium. Long-term mechanical stress may result in inflammation or localized ischemia leading to replacement fibrosis. Both histologic and MRI studies have confirmed the presence of fibrosis in these areas [30]. Furthermore, a recent hybrid PET/MRI study found FDG uptake (evidence of inflammation or ischemia) and cardiac fibrosis in these areas (and others) [32].

In addition to a substrate, a trigger is typically necessary for sustained VA. Two key theories for triggers involve abnormal mechanical stretch and Purkinje tissue. In animal models, papillary muscle traction prolonged the ventricular functional refractory period locally [33]. Given the abnormal mechanics of prolapsing leaflets in MVP, local variation in the refractory period may induce or sustain papillary muscle arrhythmias. In addition, Purkinje fibers arborize in areas adjacent to papillary muscle and in the inferior/inferolateral myocardium. They may also trigger VA in MVP patients. In one study of MVP patients undergoing ablation, all patients who had a cardiac arrest had a Purkinje origin of their arrhythmias [34]. Another study found that Purkinje potentials at the site of ablation were associated with successful ablation [34,35]. This has also been observed in a broader array of non-MVP patients. In a study of patients with papillary muscle PVCs undergoing ablation, Purkinje potentials preceded the clinical PVCs in over half of the patients [36]. It is possible that abnormal mechanical tension induces changes in the electrophysiologic function in papillary muscles and possibly the Purkinje fibers and acts as a trigger for VA in areas of cardiac fibrosis. Furthermore, the mitral regurgitation itself may lead to cardiac remodeling and further triggers for arrhythmias. 

MAD has also been associated with VA both in the presence and absence of MVP. This has led some authors to suggest that MAD is the inciting factor in the cascade that involves altered valvular mechanics, valvular degeneration, leaflet prolapse, myocardial fibrosis, and finally arrhythmias. This has been labeled the Padua hypothesis [37], and while promising, requires further research to confirm causal links. 

## 3. Natural History

The majority of patients with MVP have a benign course. However, there are subgroups at higher risk for complications. Studies are difficult to generalize due to a variety of cohorts studied. In a large study of Olmstead Countypatients, those with asymptomatic MVP had a 10-year cardiovascular morbidity and overall mortality of 30% and 19%, respectively [38]. Another study showed a similar mortality of 13% at 8 years [39]. At least half of mortality and morbidity events were felt to be related to MVP directly [38]. Aside from mortality, complications of MVP include the need for cardiac surgery, heart failure, atrial fibrillation, stroke, arterial thromboembolism, and endocarditis [28,38,40]. After 5.4 years of median follow-up, 7.8% of patients required mitral valve surgery [38].

Sudden cardiac death (SCD) and VA occur at a low rate in patients with MVP, about 0.14 per 100 patient years in community-dwelling patients [4]. Some cohorts have reported higher rates of SCD of 0.2–0.4% or higher, but these studies may have bias from referral to tertiary centers and higher-risk clinical factors [28,40,41,42]. With 8 years of follow-up, about 17% patients required ICD or VT ablation in one study of MVP patients referred to a tertiary center [39]. Although the absolute rate of SCD is low overall, the relatively high prevalence of MVP translates to a significant population at risk of SCD. MVP has been observed in about 2% of all SCD cases [4,43], but may explain as high as 10–12% in younger patients with SCD or patients with unexplained SCD [4,44].

There is a growing body of evidence identifying those with MVP at higher risk for SCD. The following section will describe known risk factors for SCD and describe an approach to risk stratification. 

## 4. Risk Stratification for Sudden Cardiac Death

### 4.1. Demographic Characteristics

Relatively little is known about the demographic factors predisposing MVP patients to SCD. Classically, SCD patients found to have MVP are younger than patients with SCD from other causes [43]. However, when studies are restricted to MVP patients only, there are conflicting results regarding whether younger or older patients are at higher risk [21,39]. Still other studies have not found any association but have mainly studied younger populations with median age in the 30s for MVP patients with SCD and control groups [29,30]. Additionally, some, but not all, studies have found that patients with MVP and SCD are more likely to be female [7,21,29,39]. Overall, it appears there is an association with younger age and female sex, as evidenced by a systematic review of MVP patients with SCD that reported a median age of 30 years with 69% of cases in females [45].

### 4.2. Electrocardiographic Abnormalities

**Figure 2:**
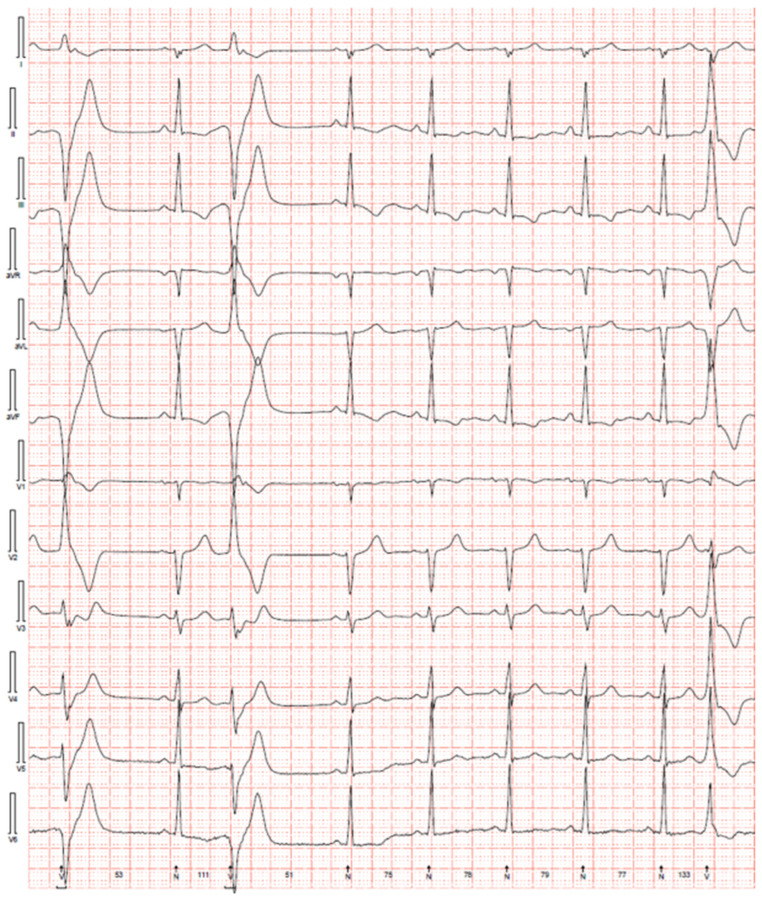
Electrocardiographic findings of arrhythmic mitral valve prolapse. Readings from 12-lead Holter monitoring demonstrating multifocal PVCs as well as T-wave inversions in leads II, III, aVF in a young otherwise healthy woman with bileaflet mitral valve prolapse, mitral annular disjunction, and a high burden of symptomatic premature ventricular contractions. The patient was inducible for sustained polymorphic VT during electrophysiology study and underwent subcutaneous implantable cardiac defibrillator (ICD) implantation.

Most patients with MVP have no clearly identifiable resting ECG abnormalities. However, several ECG abnormalities correlated with MVP and SCD have been known since the 1970s [46,47,48]. Of MVP patients who do have ventricular arrhythmias (VA, defined as premature ventricular contractions (PVCs), non-sustained ventricular tachycardia (nsVT), sustained ventricular tachycardia (VT) or ventricular fibrillation (VF)), about 65% (range 29–83%) will have T-wave inversions or biphasic T-waves, often in the inferior or inferolateral leads (Figure 2) [4,7,22,29,30,39]. MVP patients with complex VA (PVC burden of at least 5%, ventricular couplets or bigeminy, nsVT, sustained VT/VF) had an odds ratio of 2.73 for T-wave inversions compared with those without VA [49]. Some studies have also reported ST-depression and J point elevations, but it is less clear if these are more common in MVP patients with VA [7,29,39].

Individual studies produced conflicting results on whether patients with MVP with arrhythmias have longer corrected QT intervals [29,30,39,50], but a meta-analysis suggests the corrected QT interval was longer by 14.7 msec [49]. Although the corrected QT interval may be longer on average, the averages are typically not prolonged. There is not a clear threshold that predicts arrhythmias.

QT dispersion (the difference between the maximum and minimum QT intervals across all 12-leads of a single electrocardiogram) has been shown to be higher in patients with MVP than those with normal hearts and has been correlated with VA, MVP severity, and complex ventricular ectopy in MVP patients [51,52,53,54]. While these findings are thought provoking, these results are difficult to generalize given that only small, often single-center studies have shown these results. 

### 4.3. Ventricular Ectopy

Ventricular ectopy (VE) is common in patients with MVP [55]. Typically, the VE burden, complexity, and nsVT burden are higher in MVP patients with SCD [29,54]. In MVP patients with SCD, 79% had some ventricular ectopy which was complex (defined as ventricular bigeminy, multifocal PVCs, nsVT, or sustained VT/VF) in over 80% of patients (Figure 2) [4]. MVP patients with SCD almost universally had VE with a right bundle branch block morphology, but some patients with SCD may have VE with right and left bundle branch block morphology [30,56]. VE often localized to the papillary muscles and other locations [39,57]. PVCs from papillary muscles and other origins have been shown to trigger ventricular fibrillation [34,36,56]. Complex VE was often observed to be alternating between outflow tract and Purkinje/papillary muscle origins [29]. Given that PVCs are common in patients with MVP, the presence of PVCs alone is not a high-risk marker. As noted above, complex VE, NSVT, and higher PVC burden (perhaps ≥5%) [39] appear to correlate with increased risk. 

### 4.4. Mitral Valve Characteristics

**Figure 3:**
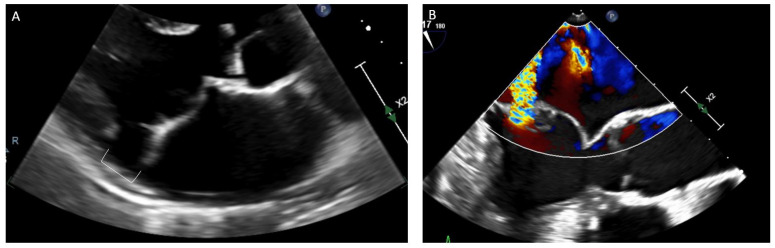
Echocardiographic findings of arrhythmic mitral valve prolapse and mitral annular disjunction. (**A**) Transthoracic echocardiography view of bileaflet prolapse seen with mitral annular disjunction (bracket) in the parasternal long axis view. (**B**) Transesophageal echocardiography view of mitral valve prolapse with color Doppler showing mitral regurgitation in a mid-esophageal long axis view.

Several anatomic characteristics of the mitral valve (MV) leaflets themselves have been associated with VA and SCD (Figure 3). The presence of classic MVP with thickened and redundant MV leaflets (Barlow’s disease) carries a higher risk for SCD than those with non-classic MVP [7,28,39]. The extent of leaflet prolapse has been correlated with SCD with highest risk for bileaflet prolapse [29,39,58]. One meta-analysis reported that MVP patients with bileaflet prolapse had an odds ratio of 1.65 (*p* value < 0.001) for complex VA (PVC burden of at least 5%, ventricular couplets/bigeminy, nsVT, sustained VT/VF) compared with segmental or single leaflet prolapse [49]. Longer mitral valve leaflet length (either anterior, posterior, or both) has been correlated with SCD but specific numerical cut offs are not clear [30,39,49,54,59]. An older study reported a high rate of SCD in patients with flail leaflets [41], but an association between flail and SCD was not reproduced with a more recent study [39]. A larger mitral valve annular diameter was associated with the arrhythmias, but the mean difference in annular diameter was relatively small [21]. 

The degree of regurgitation appears to be associated with SCD, although patients with minimal regurgitation may still experience SCD [39,60]. The association with severity of regurgitation may also be reduced when controlling for other factors such as LV remodeling, which may also increase risk [39]. There are inconsistent findings related to left atrial/ventricular dilation, LV ejection fraction and arrhythmias [29,30,39,41,60,61].

### 4.5. Novel Echocardiographic Findings

Advances in echocardiographic techniques have led to the identification of several abnormalities associated with VA. As described below, these advances include the mitral annular tissue Doppler velocity, global longitudinal strain, mechanical dispersion, and post-systolic shortening. While these new imaging findings are promising, most have only been demonstrated in small studies and require further validation prior to mainstream utilization. 

Pickelhaube sign: In one study of 21 patients, a spike in the mitral annular tissue-Doppler velocity of >16 cm/sec was associated with VA [62]. This spike was most often detected in the lateral and posterolateral annulus [63]. The graphical spike resembled the “Pickelhaube” helmet of the historical German military, and the authors termed this finding the Pickelhaube sign. 

Speckle-tracking echocardiography: Changes in speckle-tracking echocardiography imaging can precede more classic changes in cardiac function (e.g., LV ejection fraction) and have been associated with arrhythmic outcomes in hypertrophic cardiomyopathy [64]. Patients with MVP have a lower global longitudinal strain (GLS) than controls (an absolute value of 16 or lower is considered abnormal strain), and arrhythmic MVP patients have even lower GLS than non-arrhythmic MVP [21,61,65]. Some studies have suggested abnormal regional strain in the basal left ventricle, but a consistent association with arrhythmic MVP has not been proven [66,67].

Mechanical dispersion is defined as the standard deviation of the time to peak longitudinal strain in all LV segments. A larger number correlates with mechanical dyssynchrony. Increased mechanical dispersion correlates with myocardial fibrosis and arrhythmic risk in hypertrophic cardiomyopathy and ischemic cardiomyopathy [68,69]. Arrhythmic MVP patients have higher mechanical dispersion than MVP patients without arrhythmias [21,22].

Other speckle-tracking echocardiography techniques such as circumferential or radial strain, and post-systolic shortening may be associated with MVP with VA, but further validation is required [61,67,70].

### 4.6. Cardiac Magnetic Resonance 

**Figure 4:**
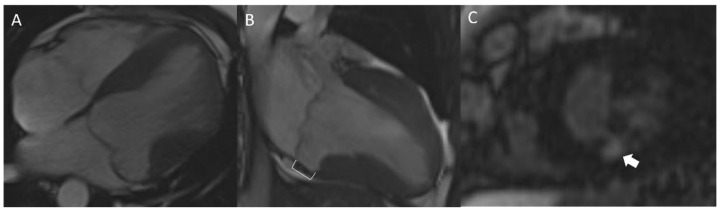
Cardiac magnetic resonance findings of arrhythmic mitral valve prolapse and mitral annular disjunction. (**A**) Bileaflet prolapse seen in a view analogous to apical 4 chamber view. (**B**) Mitral annular disjunction (bracket) where the mitral valve hinge point separates from the basal left ventricular myocardium and has systolic excursion into the left atrium. (**C**) Late gadolinium enhancement of a papillary muscle (arrow) seen in a view analogous to parasternal short axis.

Cardiac magnetic resonance (CMR) is a powerful technique to assess cardiac structure and function, including cardiac fibrosis. In addition to evaluation of valve structure and regurgitation, contrast enhanced CMR with delayed imaging can demonstrate late gadolinium enhancement (LGE), a marker for replacement myocardial fibrosis (Figure 4). LGE is significantly more common in MVP patients than controls [42]. Depending on the cohort studies, between 28 and 93% patients with MVP had LGE [30,42,71,72,73,74]. LGE is most commonly seen in the basal inferolateral wall and papillary muscles and less commonly in the myocardium adjacent to papillary muscles or other locations [30,71]. Arrhythmic MVP is more strongly associated with LGE than non-arrhythmic MVP [30,71], with a relative risk of 4.38 (*p* = 0.001) in a meta-analysis [49]. Areas of LGE correlate with histologic scar in pathologic specimens from patients who suffered SCD and correlate with the origins of VA identified during electrophysiologic studies [30,75,76]. 

A recent histopathologic analysis of MVP with SCD also suggested that MVP patients may have a more diffuse type of fibrosis compared with controls [75]. This diffuse myocardial fibrosis can be identified with T1 mapping or with increased myocardial extracellular volume [72,77]. Taken together, a growing body of evidence suggests histologic fibrosis, both diffuse and focal to the papillary muscles and inferolateral wall, strongly correlate with arrhythmias and can be accurately assessed non-invasively with CMR utilizing post-contrast LGE sequences and other sequences focused on diffuse fibrosis.

### 4.7. Mitral Annular Disjunction and Annular Curling

As described above, MAD is defined as mitral annular detachment from the basal LV myocardium with an abnormal systolic excursion of the mitral valve hinge point into the left atrium (Figure 4). It is present in about 33% of patients with MVP. MAD is also associated with annular curling. MAD has been associated with VA both in patients without MVP [13] and those with MVP [13,17,21,23]. A meta-analysis reported MVP patients with MAD had a higher risk of VA, with a risk ratio of 1.90 (*p* < 0.0001) [49]. 

### 4.8. Approach

**Figure 5:**
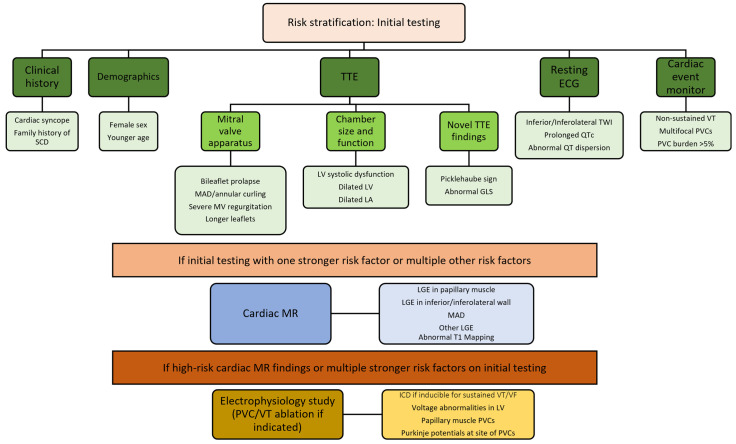
Clinical approach to risk stratification for sudden cardiac death or complex ventricular arrhythmias in MVP patients with clinical concern for arrhythmias. Most patients should undergo initial baseline testing with history, transthoracic echocardiography, and resting 12-lead ECG. Cardiac monitoring should be considered in those with a clinical concern for arrhythmias or if the presence of ventricular arrhythmias on monitoring would significantly modify risk categorization. If testing is abnormal or multiple risk factors are seen, cardiac MR can be considered. If there is an abnormal cardiac MR or very high-risk initial history and testing, we utilize a shared decision-making approach to electrophysiology study and possible implantable cardiac defibrillator. Premature ventricular contraction and/or ventricular tachycardia ablation can be considered as well if indicated (see text). TTE: transthoracic echocardiogram; ECG: electrocardiogram; SCD: sudden cardiac death; TWI: T-wave inversions; QTc: corrected QT interval; VT: ventricular tachycardia; PVC: premature ventricular complex; MAD: mitral annular disjunction; MVP: mitral valve prolapse; LV: left ventricle; LA: left atrium; GLS: global longitudinal strain; cardiac MR: cardiac magnetic resonance imaging: LGE: late gadolinium enhancement.

Given limited multicenter prospective data, arrhythmic risk stratification for MVP must be individualized and cost-effectiveness should be considered. Our specific approach is outlined in Figure 5. For those with historical and physical exam findings (mid-systolic click with apical regurgitation murmur), a detailed history, physical exam, and echocardiogram are typically the first steps. Once MVP is confirmed, images should be specifically reviewed for evidence of MAD and annular curling, extent of leaflet prolapse, chamber size and function, strain, and tissue Doppler velocities. A resting electrocardiogram should be performed in all patients with MVP. Cardiac event monitors should be considered in patients with clinical symptoms of arrhythmia (palpitations, syncope, lightheadedness, etc.). Based on our expert opinion, for those with clinical concern for arrhythmias and risk factors (see text) on initial evaluation leading to intermediate to high risk concern, a cardiac MRI and genetic testing are frequently recommended. With the paucity of data, however, it is important to combine objective testing with the overall concern for arrhythmia based on the history (symptoms, prior clinical events, and family history). If cardiac MRI is abnormal or initial history and testing is very high risk, an electrophysiology study may be indicated (see management). Cardiac MRI and cardiac monitors may not be necessary in all patients but may help guide decision making as an adjunct.

## 5. Management

In general, MVP patients with significant valvular heart disease and arrhythmias are treated according to standard guidelines for arrhythmias, valvular heart disease, and heart failure [78]. To date, there are no specific guidelines or consensus statements on the management of arrhythmias related to MVP. 

### 5.1. Electrophysiology Study

The role of electrophysiology study (EPS) in risk stratification for patients with MVP is unclear. During an EPS, ventricular burst pacing or ventricular extra-stimulus testing can be utilized to assess the risk of future sustained VA and the potential need for an implantable cardiac defibrillator (ICD). As discussed above, there are multiple known risk factors for increased risk of ventricular arrhythmias, although formal indications for EPS in MVP patients do not exist. One study of MVP patients undergoing ablation showed that 5/5 patients of those that had sustained VA after an ablation experienced sustained VT during EPS prior to first ablation [79]. This suggests that the inducibility of sustained VA may be helpful in accurately predicting those at higher risk for developing cardiac arrest. 

### 5.2. Anti-Arrhythmic Drug Therapy

There is relatively little data regarding the efficacy of anti-arrhythmic drugs for VA specifically related to MVP. Due to their relative safety, medical therapy is often first line. PVC suppression by anti-arrhythmic drug therapy (beta and calcium channel blockers, other anti-arrhythmic drugs) in one small study was not efficacious [57]. There is no clear evidence suggesting any particular agent or class is more efficacious beyond standard guidelines for non-MVP-related VA.

### 5.3. Device Therapy

The use of ICDs should follow standard guidelines. Patients with sustained ventricular tachycardia or fibrillation should be considered for secondary prevention ICD. The role of primary prevention ICD is unclear. As mentioned above, an electrophysiology study may be helpful for risk stratification, but currently there are no robust data to support primary prevention ICD in any other specific populations and no formal risk calculators. If arrhythmic MVP is being considered, a cardiac MRI prior to device implantation may be helpful.

### 5.4. Catheter Ablation

Catheter ablation in patients with MVP is typically carried out for frequent symptomatic ventricular ectopy, sustained ventricular fibrillation/tachycardia (including PVC triggered), or recurrent appropriate ICD discharges refractory to anti-arrhythmic drug therapy. Of note, in one prior series where the ablation of PVCs triggering VF was performed, one patient had incessant VF in the first 24 h post ablation [34].

As discussed above, MVP-related PVCs often originate from the papillary muscles and from Purkinje fibers. Other sources include the mitral valve annulus, right ventricular outflow tract, septum, LV lateral wall, or other locations (LV apex, tricuspid valve annulus, left ventricular outflow tract most common) [34,57,79,80]. A 12-lead Holter is helpful in localizing clinical PVCs prior to the procedure. Cardiac MRI may also help to localize substrate for arrhythmias and guide ablation. Sedation is typically kept as light as possible to reduce the suppression of PVCs and anti-arrhythmic drugs are usually washed out prior to ablation. LV mapping and ablation can be performed either via retrograde aortic or transseptal approach. Abnormal bipolar or unipolar voltage are often noted to involve the inferior and inferolateral walls [56]. 

Given the predisposition to papillary muscle arrhythmias, ablation can be challenging in MVP patients [81]. The anatomic structure of the papillary muscles may allow a single focus with multiple exit points and produce multiple QRS morphologies. Ablation at a single focal papillary muscle site with an excellent pace map may be insufficient. Instead, patients may require multiple lesions along the papillary muscle [82]. Given these difficulties, the primary strategy is often activation mapping of PVCs occurring spontaneously or induced (for example from ventricular or atrial burst pacing or with isoproterenol). The goal is to identify the earliest local bipolar activation time compared with a reliable QRS on the surface ECG and a QS unipolar tracing. Several studies have shown Purkinje potentials at the sites of successful PVC ablation in a significant proportion of patients [34,56]. Patients with papillary muscle-related arrhythmias have higher recurrence rates and ablations are less frequently effective compared with other sites of origin [83].

**Figure 6:**
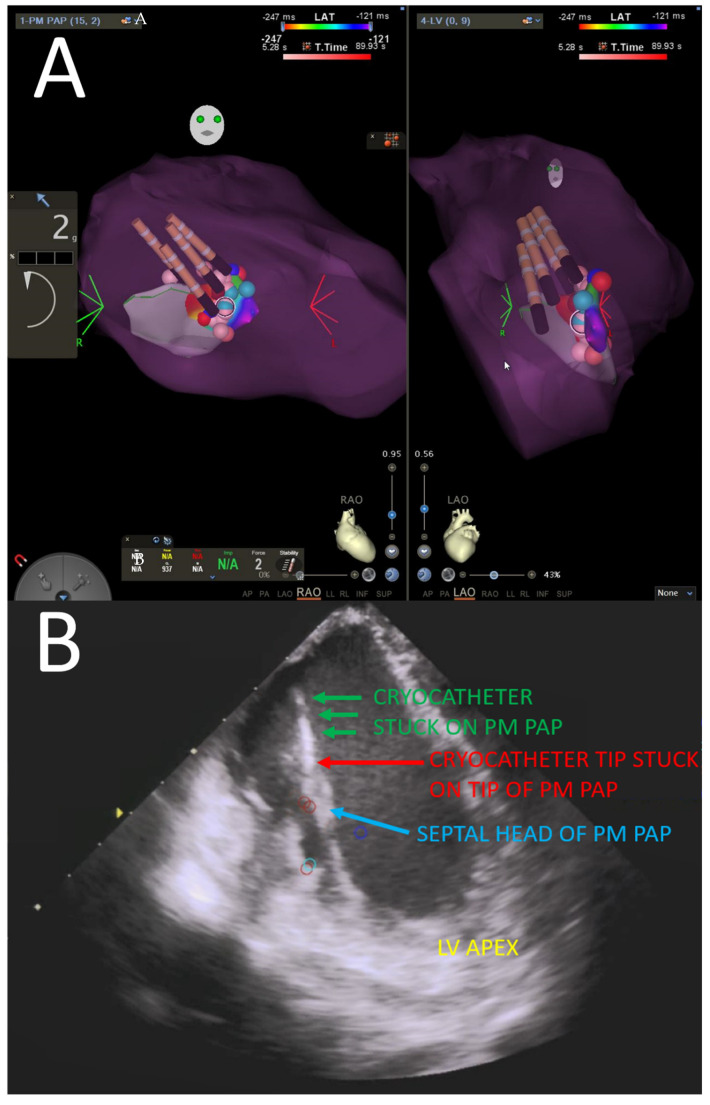
Papillary muscle ablation utilizing cryo-energy and intracardiac echocardiography. (**A**) Electroanatomic map highlighting cryo-energy lesions to a papillary muscle. (**B**) Intracardiac echocardiography is a useful adjunct to confirm good contact and stability of catheter to papillary muscle during ablation. PM PAP: posteromedial papillary muscle, LV: left ventricle.

For ablation, the papillary muscle structure makes catheter engagement and stability more difficult. Steerable sheaths may be helpful. Intra-cardiac echocardiography (ICE) is a useful adjunct to confirm anatomic position and stability. High energy is often necessary to create adequate injury given the poor contact. In papillary muscle PVC cases refractory to radiofrequency ablation, cryoablation should be considered as it may result in improved catheter stability during ablation (Figure 6) [84,85,86].

PVC ablation has been shown to reduce ventricular ectopy burden, prevent ICD shocks, and improve systolic function (in patients with PVC cardiomyopathy) [34,35,56]. Improvement in VE burden and the non-inducibility of ventricular tachycardia can be achieved in 60–86% of patients acutely [34,35,56,79,80]. Long-term, however, about 20–40% may have a recurrence of arrhythmia or require repeat ablation [34,79,80]. One small study suggested that those with multifocal VE were more likely to require repeat ablation [79]. Repeat ablation typically identified arrhythmias with new foci compared with index ablations [34,79].

### 5.5. Surgical Intervention

Surgical indications for mitral valve repair in the setting of MVP depend on the severity of mitral regurgitation; the role of surgery and intraoperative ablation for the prevention of SCD is unknown. Historically, multiple case reports and series, typically with fewer than ten patients with MVP and VA, showed a reduction in VA after mitral valve surgery, even when the regurgitation was insignificant [87,88,89]. More recently, results regarding the effect of mitral valve surgery on ventricular ectopy have been mixed [90,91]. For MAD, medically managed patients had significantly worse arrhythmia-free survival compared with MAD-free controls, but MAD patients undergoing mitral surgery had similar arrhythmia-free survival to controls [23]. Surgical cryoablation, often utilized for atrial fibrillation treatment during cardiac surgery, also holds some promise for PVC treatment. One recent study of three patients with MVP and papillary muscle PVCs undergoing cardiac surgery for severe mitral regurgitation also underwent intra-operative visually guided (no electro-anatomic mapping) cryoablation to the papillary muscle base and tip [92]. With short-term follow-up, there was a significant reduction in PVCs. Overall, given the lack of prospective data, firm conclusions on the effect on mitral valve surgery or surgical cryoablation are difficult to draw but warrant further study.

## 6. Conclusions

Only a small proportion of patients with MVP and MAD will suffer from VA, but given the relative prevalence of MVP and MAD worldwide, it is a significant cause of SCD. Over the last decade, retrospective research studies have identified risk factors for SCD, including abnormalities in electrocardiography, echocardiography, and cardiac MRI. Initial research suggests that electrophysiologic studies can accurately predict the need for ICD in well-selected patients and that catheter ablation of VA is efficacious when indicated. Future prospective research is needed to further delineate optimal methods for SCD risk stratification and treatment.

## Data Availability

Primary data in the form of electrocardiogram, imaging, or ablation data is available on request with restrictions for patient privacy.
